# 110. A Phase 3, Multicenter, Double-blind, Randomized Clinical Trial to Evaluate the Efficacy and Safety of Ceftolozane/Tazobactam Plus Metronidazole Versus Meropenem in Chinese Participants With Complicated Intra-abdominal Infections

**DOI:** 10.1093/ofid/ofab466.110

**Published:** 2021-12-04

**Authors:** Yihong Sun, Jia Fan, Gang Chen, Xiaofei Chen, Xiaoling Du, Ye Wang, Hui Wang, Fang Sun, Matthew G Johnson, Mekki Bensaci, Jennifer A Huntington, Christopher Bruno

**Affiliations:** 1 Zhongshan Hospital, Fudan University, Shanghai, Shanghai, China; 2 The First Hospital of Kunming, Kunming, Yunnan, China; 3 MSD, China, Shanghai, Shanghai, China; 4 Merck & Co., Inc., Kenilworth, NJ

## Abstract

**Background:**

In China, the prevalence of infections due to multidrug-resistant gram-negative bacteria is high and additional treatment options for complicated intra-abdominal infections (cIAI) are needed. This study compared the efficacy and safety of ceftolozane/tazobactam (C/T) + metronidazole (MTZ) versus meropenem (MEM) + placebo (pbo) for the treatment of cIAI in adult Chinese participants.

**Methods:**

This was a phase 3, double-blind study conducted at 21 centers in China (NCT03830333). Participants aged 18-75 years with cIAI requiring surgical intervention within 24 hours of study drug administration were stratified by site of infection and randomized 1:1 to receive 1.5 g C/T (1 g ceftolozane and 0.5 g tazobactam) + 0.5 g MTZ administered intravenously (IV) every 8 hours (q8h) or 1 g MEM + pbo administered IV q8h for 4-14 days. The primary endpoint was clinical cure at test of cure (TOC) in the clinically evaluable (CE) population. Secondary endpoints included rates of clinical cure, per-participant microbiologic response, per-pathogen microbiologic response, and adverse events (AE). Non-inferiority for clinical cure at TOC in the CE population was confirmed if the lower bound of the 2-sided 95% CI for the between-treatment difference in the clinical cure rate was larger than −12.5%.

**Results:**

A total of 134 participants were randomized to each treatment group. Demographics and baseline characteristics were generally well balanced between treatment groups (Table 1). The median (range) age in the ITT population was 50 (18-75) years and 61% were men. The most frequent sites of infection were the appendix (C/T + MTZ, 50.0%; MEM + pbo, 49.3%) and gallbladder (C/T + MTZ, 27.6%; MEM + pbo, 29.1%). Overall, the most frequently isolated pathogens were *Escherichia coli* (61.4%) and *Klebsiella pneumoniae* (17.3%); few anaerobes were isolated (Table 1). C/T + MTZ was non-inferior to MEM + pbo for clinical cure in the CE population (C/T + MTZ, 95.2%; MEM + pbo, 93.1%; difference, 2.1% [95% CI, −4.7% to 8.8%]). Results for key secondary endpoints were comparable between treatment groups (Table 2). Rates of AEs were generally similar between treatment groups (Table 3).

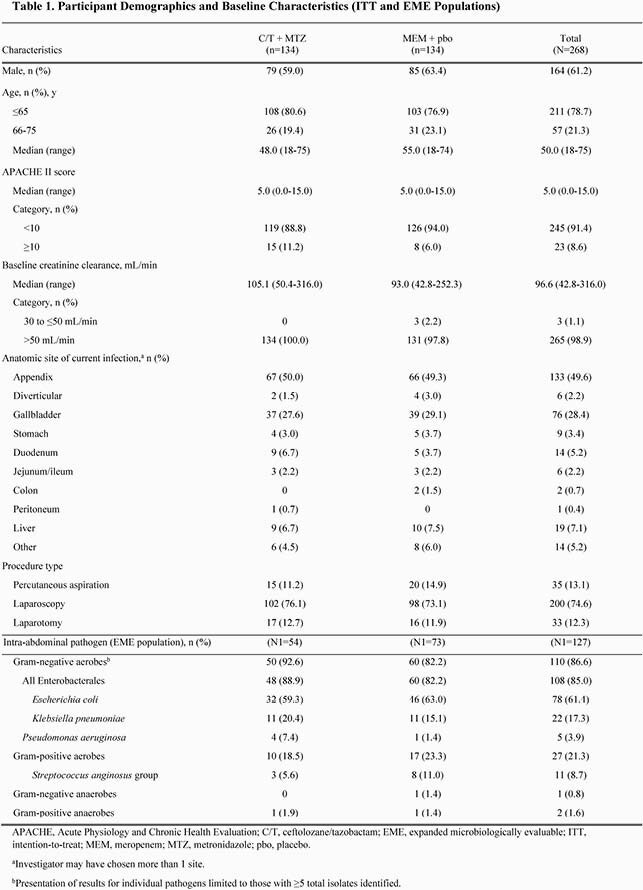

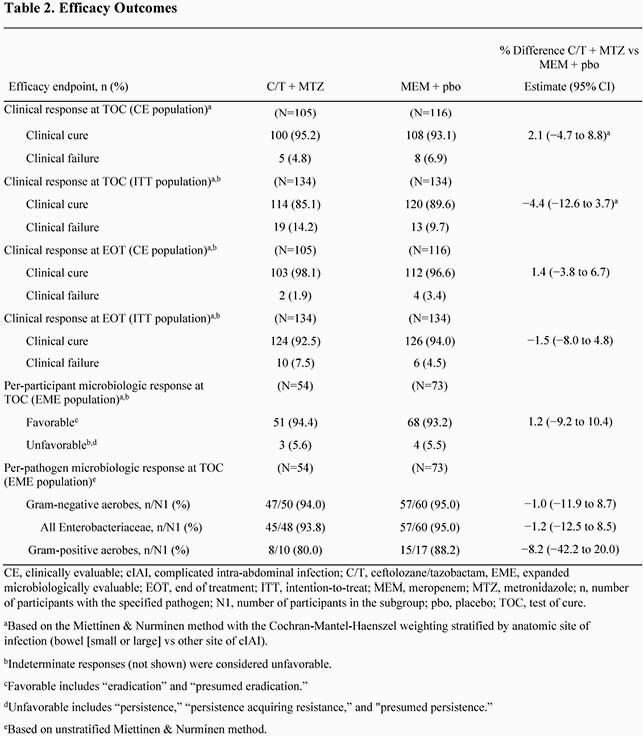

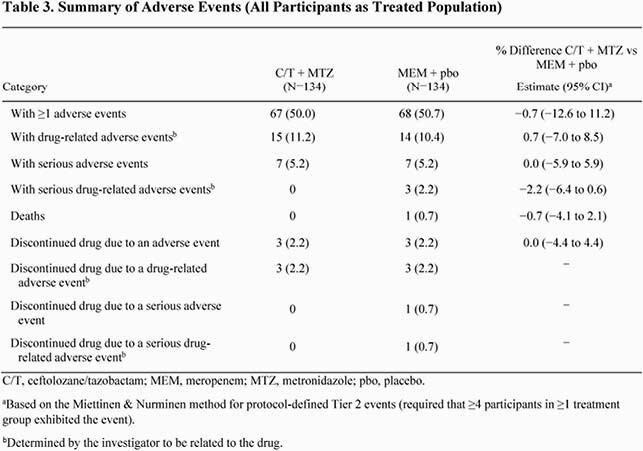

**Conclusion:**

C/T + MTZ was non-inferior to MEM + pbo in the treatment of adult Chinese participants with cIAI and demonstrated a favorable safety profile.

**Disclosures:**

**Xiaofei Chen, n/a**, **MSD, China** (Employee) **Xiaoling Du, n/a**, **MSD, China** (Employee) **Ye Wang, n/a**, **MSD, China** (Employee) **Hui Wang, n/a**, **MSD, China** (Employee) **Fang Sun, n/a**, **MSD, China** (Employee) **Matthew G. Johnson, MD**, **Merck Sharp & Dohme Corp., a subsidiary of Merck & Co., Inc., Kenilworth, NJ, USA** (Employee) **Mekki Bensaci, PhD**, **Merck Sharp & Dohme Corp., a subsidiary of Merck & Co., Inc., Kenilworth, NJ, USA** (Employee) **Jennifer A. Huntington, PharmD**, **Merck Sharp & Dohme Corp., a subsidiary of Merck & Co., Inc., Kenilworth, NJ, USA** (Employee) **Christopher Bruno, MD**, **Merck Sharp & Dohme Corp., a subsidiary of Merck & Co., Inc., Kenilworth, NJ, USA** (Employee)

